# Titanium elastic nailing versus locking compression plating in school-aged pediatric subtrochanteric femur fractures

**DOI:** 10.1097/MD.0000000000011568

**Published:** 2018-07-20

**Authors:** Yunlan Xu, Jingxia Bian, Kaiying Shen, Bin Xue

**Affiliations:** a Department of Pediatric Orthopedics; b Department of Anesthesiology, Shanghai Children's Medical Center, Affiliated to Shanghai Jiaotong University School of Medicine, Shanghai, China.

**Keywords:** internal facture fixation, locking compression plate, open reduction, pediatric, subtrochanteric fractures, titanium elastic nail

## Abstract

The treatment of children between 5 and 12 years of age suffering from subtrochanteric femoral fracture is challenging. The optimal choice of internal fixation for these patients is controversial. The purpose of this study is to compare the outcomes and complications of titanium elastic nail and open reduction with plate fixation of subtrochanteric femur fractures in school-aged children.

A total of 67 children aged 5 to 12 years with subtrochanteric femur fractures treated with titanium elastic nails or open plating were identified at our institution from January 2007 to December 2017. We retrospectively compared 39 children treated with titanium elastic nails with 28 children treated with open reduction and plate fixation. The data included age, sex, body weight, fracture pattern, operation time, blood loss, and length of hospitalization. The follow-up investigations included radiograph of pelvis, bilateral hip range of motion, bilateral femoral neck shaft angle, and length of lower extremity. The outcomes were classified according to Flynn classification as excellent, satisfactory, or poor. All the demographic characteristics were compared with statistical analyses.

All 67 fractures united properly. No major postoperative complications were noted in both groups. No significant difference was found between the titanium elastic nail and open plating groups in terms of sex, fracture pattern, and length of hospitalization. We noted a significant difference between 2 groups in terms of age, weight, operation time, and blood loss. In total, we observed 24 excellent and 15 satisfactory results in the titanium elastic nail group, and 19 excellent results and 9 satisfactory results in the open plating group. There was no significant statistical difference between involved and uninvolved side of hip regarding range of motion and femoral neck shaft angle in both groups.

Titanium elastic nail and pediatric hip plate fixation represent safe and effective methods in the treatment of subtrochanteric fractures in school-aged children. Titanium elastic nail internal fixation is a minimal invasive and simpler technique and suitable for young children of lower body weight. Open plate fixation is a more rigid fixation associated with a lower complication rate.

## 1 Introduction

Fractures of the proximal third of the femur, particularly in the subtrochanteric region, are at a higher risk for complications. Correct fracture reduction is more difficult to obtain or maintain because of their special anatomical position.^[1–3]^ Rigid intramedullary nailing and locking compression plating are the preferred treatment in adolescents.^[4,5]^ Few studies focused on the treatment of the school-aged children or young adolescents between 5 and 12 years of age suffering from subtrochanteric femur fracture.^[6]^ The optimal choice of internal fixation for these patients is discussed controversially. The purposes of this study are to (1) compare radiological/clinical outcome of titanium elastic nail (TEN) and plate fixation in subtrochanteric femur fractures in school-age children, (2) and to analyze any possible complications.

## 2 Materials and methods

A consecutive series of patients aged 5 to 12 years with traumatic subtrochanteric femur fractures were retrospectively compared. We compared treatment using TENs; Synthes; Synthes Biomaterials, Oberdorf, Switzerland) to open plating internal (OPI) fixation using locking compression plate (LCP; Synthes; Synthes Biomaterials, Oberdorf, Switzerland) between January 2007 and December 2017 in our institution. Informed consent was obtained and institutional review board approval was acquired.

A subtrochanteric femur fracture was defined as a fracture that was located within 10% of the total femur length below the lesser trochanter according to Pombo and Shilt.^[7]^ The first available postoperative full-length anteroposterior (AP) femur radiograph was used to determine the total length of the femur, which was defined as the distance between the top of the femoral head and the medial femoral condyle. Next, the distance between the inferior aspect of the lesser trochanter and the fracture site was measured. If this distance was ≤10% of the total length of the femur, the fracture was classified as subtrochanteric. Pathologic fractures, fractures in patients with osteogenesis imperfecta, or fractures in patients suffering from neuromuscular disorders were excluded.

The data collected included age, weight, sex, fracture pattern, method of fixation, postoperative immobilization, length of hospitalization, time to radiographic union, time of operation, bleeding during the operation, bilateral hip ranges of motion (ROM), and femoral neck-shaft angle (NSA) between involved and uninvolved side after surgery. The radiographic data included AP view of pelvis, AP and lateral views of femur, preoperatively and postoperatively. Fracture patterns were classified as length stable or length unstable. Length-stable fractures were transverse and short oblique. Length-unstable fractures were comminuted and long oblique fractures, where the length of the obliquity was at least twice the diameter of the femoral shaft at that level.^[3]^ Radiographic union was defined as bridging callus across at least three of the four cortices at the fracture site seen both on AP and lateral radiographs of the femur.

The final functional outcomes evaluated by using Flynn scoring system^[1]^ were classified into excellent, satisfactory, or poor based on residual leg-length inequality, fracture malalignment, pain, complications, and unplanned surgery for the treatment of complications (Table 1).

**Table 1:**
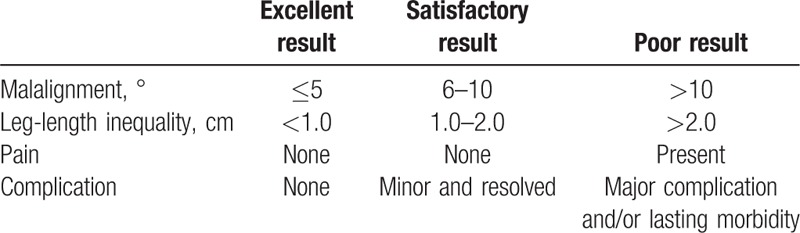
Titanium Elastic Nails Outcome Scoring System by Flynn et al^[1]^.

## 3 Postoperative care

Spica casting was used in patients of the TEN group for 6 weeks and long-leg casting was used in patients of the OPI group for 2 weeks after surgery. All patients started motion exercises after cast removal. In the TEN group, patients were allowed to bear toe-touch weight when the radiographic union occurred and permitted to gradually bear more weight based on clinical and radiographic evidence of healing. Patients in the OPI group started toe-touch weight bearing with crutches at approximately 4 weeks postoperatively. These patients were allowed to bear no limited weight only when radiographic evidence of healing had been achieved.

## 4 Statistical methods

SPSS 13.0 (SPSS Inc., Chicago, IL) was used for all statistical analyses. We compared the demographic characteristics between the 2 treatment groups using the χ^2^ tests for categorical variables and analyses of the Mann–Whitney *U* tests for continuous variables. The Wilcoxon rank test was used to compare the bilateral ROM and NSA, and *P* values <.05 were considered significant.

## 5 Results

**Figure 1:**
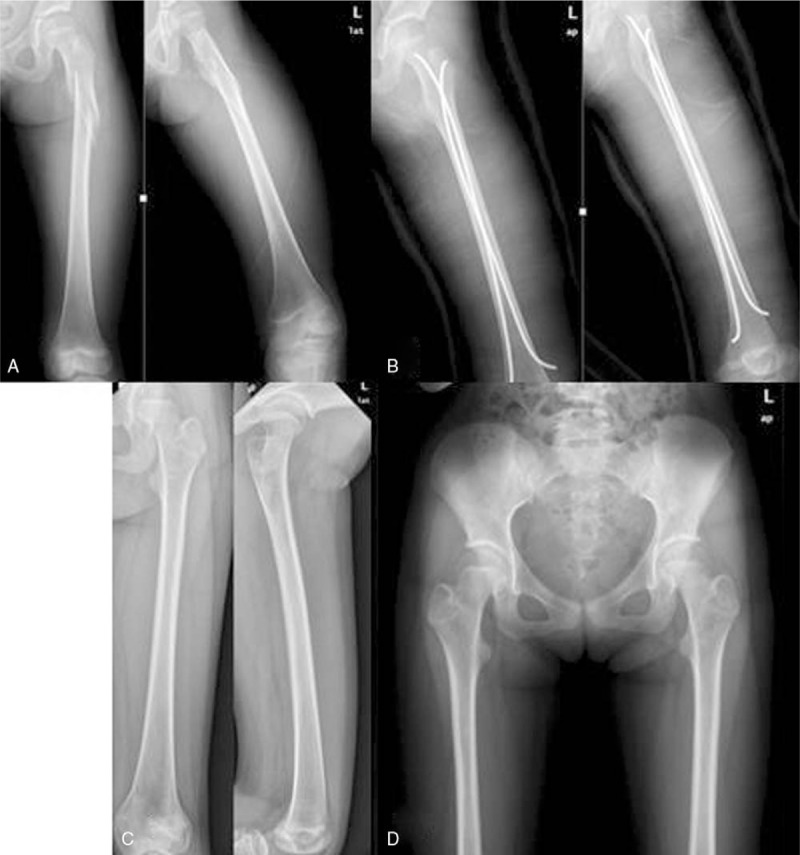
A 7.5-year-old male fell down from a height of 1 m. (A) AP and lateral radiographs of the left femur with long oblique unstable subtrochanteric fracture. (B) Immediate postoperative radiographs after closed reduction and fixation with TENs. (C) Six months after surgery, the fracture had good healing radiographically. (D) AP view of pelvis showed a symmetric normal NSA between involved and uninvolved limb after 38 months.

**Figure 2:**
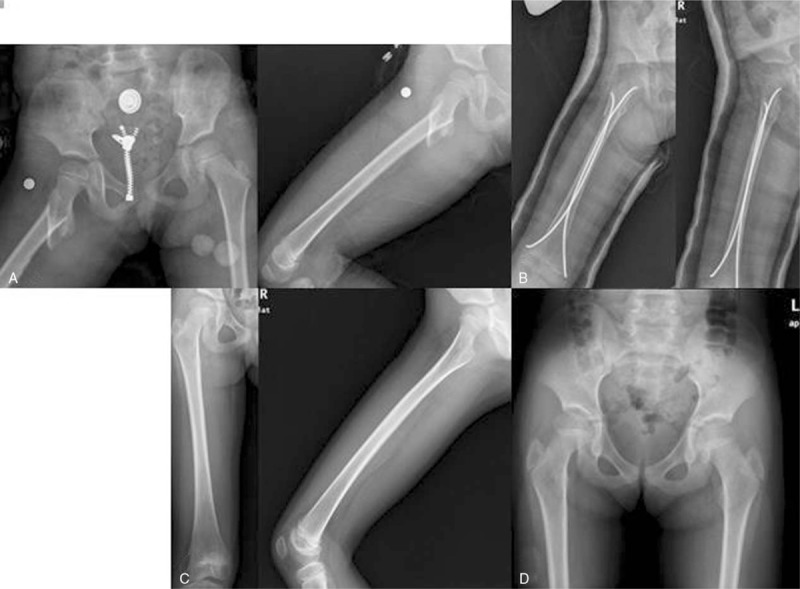
A 7.6-year-old female suffered traffic accident. (A) Preoperative radiographs of the right femur with short oblique stable subtrochanteric fracture. (B) Immediate postoperative radiographs after closed reduction and fixation with 2 nails. (C) The bone healed well 6 months after surgery. (D) The patient showed a normal NSA (as on the uninvolved side) and a 1.5 cm limb length discrepancy with overgrowth of involved side.

**Figure 3:**
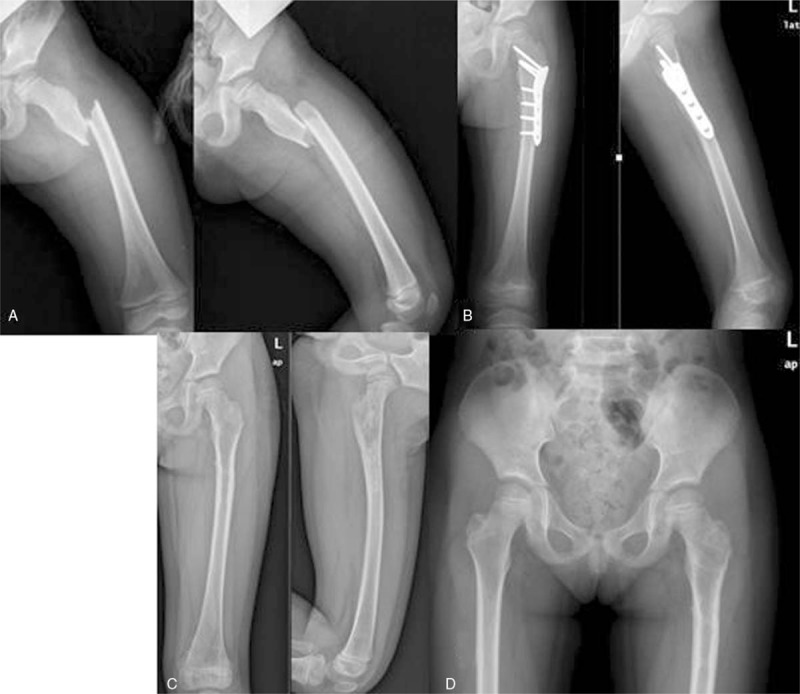
A 6.8-year-old boy sustained an injury when falling from height. (A) Anteroposterior and lateral views of the left femur showed a short oblique stable subtrochanteric fracture. (B) The fracture was fixed with pediatric hip plate by open reduction. (C) Eight months after surgery, the fracture healed well and plate and screws were removed. (D) The patient showed a symmetric NSA. We noted a limb length inequality of -1 cm.

**Figure 4:**
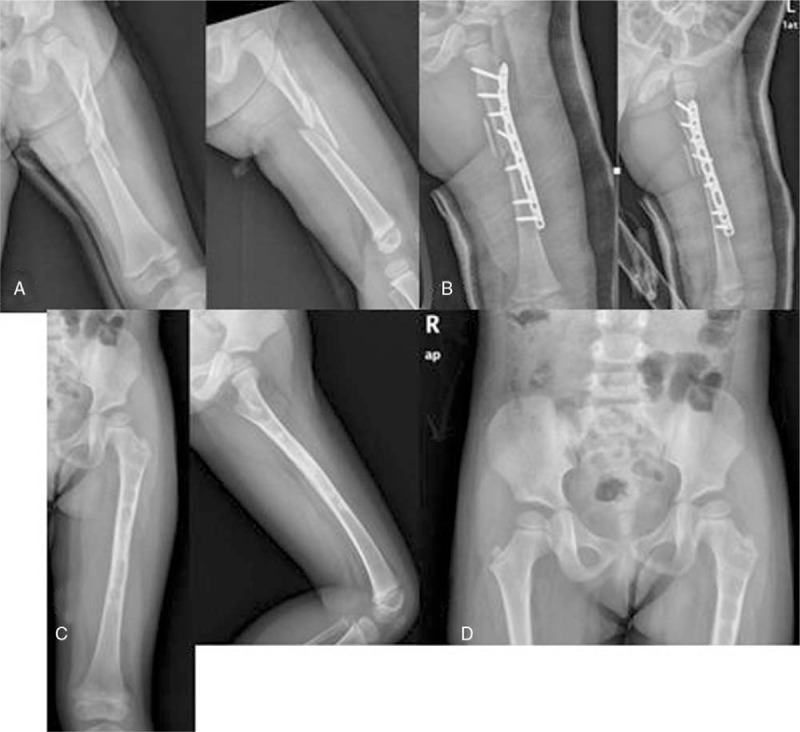
A 5.5-year-old boy suffered traffic accident. (A) AP and lateral radiographs of the right femur with comminuted unstable fracture of thesubtrochanteric region to shaft. (B) The patient was treated by open reduction with locking compression plate fixation. (C) Ten months after surgery, the fracture showed good healing radiographically and internal fixation was removed. (D) AP view of pelvis showed a symmetric NSA and no limb length inequality.

A total of 67 patients (40 boys and 27 girls) met the inclusion criteria. Thirty-nine patients were treated with TEN method (Figs. 1 and 2) and 28 patients were treated with OPI method (Figs. 3 and 4). The average age of the patients was 8.3 ± 2.0 years (range, 5–11.9 years) and the average weight was 35.5 ± 5.9 kg (range, 25.3–49.6 kg). There were 28 cases of length stable fracture and 11 cases of length unstable fracture in the TEN group. Correspondingly, 20 cases of stable fracture and 8 unstable fractures were in the OPI group. The average operation time of group TEN was 41.2 minutes; the average blood loss of surgery was 8.2 mL; and the average operation time of OPI group was 98 minutes and the average blood loss was 70 mL. The TEN group was hospitalized on an average of 5.7 days, while the OPI group was hospitalized on an average of 6.5 days. The average follow-up time was 28.5 months (range, 16–42 months).

We noted no statistically significant difference between the 2 groups in terms of sex, fracture pattern, and length of hospitalization. However, there was a significant difference in age, weight, time of operation, and bleeding during the operation (Table 2).

**Table 2:**
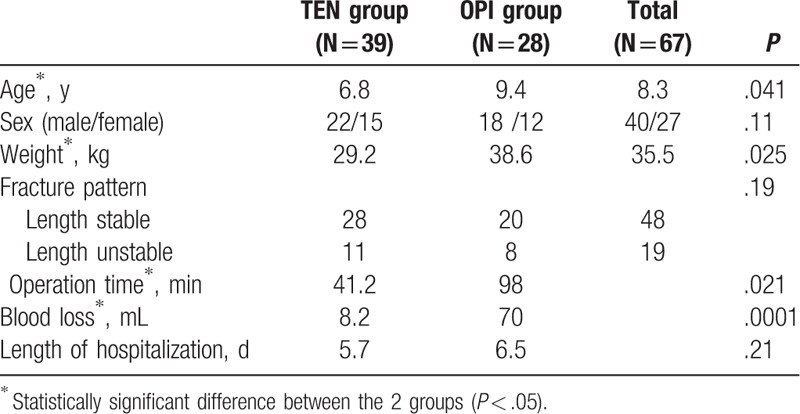
Demographic data.

At the final follow-up, all patients were able to walk without limping and had full symmetric range of motion in the hip joint with no difference to the uninvolved side in both groups (*P* > .05) (Table 3). According to the Flynn outcome scores, excellent results were demonstrated in 62% of patients in the TEN group and in 68% of patients in the OPI group. Thirty-eight percent of patients in the TEN group and 32% of patients in the OPI group had results as satisfactory. No poor results were observed in both groups (Table 4).

**Table 3:**
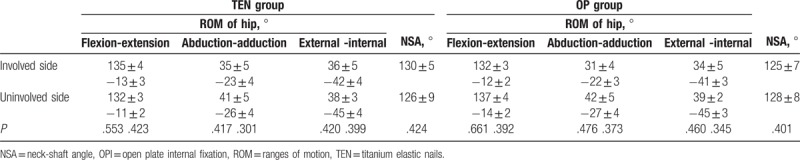
Comparison of hips ROM and NSA between involved and uninvolved side after operation.

**Table 4:**
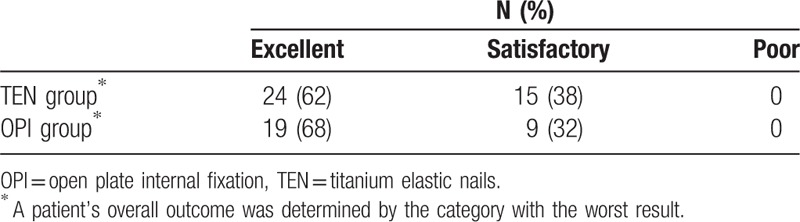
Flynn outcome scores.

The complication rates in both groups were low and similar (18% vs 14%, *P* = .073). The most common complication in the TEN group was pain from prominent nail end (3 patients), which was relieved after using a knee immobilizer. Two patients with 6° to 10 ° of fracture malalignment at the time of radiographic union were found and the malalignment had remodeled at the time of final follow-up. Two patients, who were treated with TEN, demonstrated leg-length inequality where the affected limb was 1.5 cm longer (Fig. 2) or 1 cm shorter (Fig. 3). Among the patients who had undergone open plating, 1 had 6 ° of varus at the time of fracture union and 3 had leg length inequality where the involved limb was longer (ranged from 1 to 2.5 cm) than the other side. No patient underwent unplanned surgery for complications in both groups.

## 6 Discussion

The treatment of pediatric subtrochanteric femur fractures is discussed controversially. Especially, the method of fixation with different implants is still an open question. We retrospectively compared TENs with open plating for the treatment of subtrochanteric femur fractures in school-aged children. The results showed that both TEN and OPI method can obtain good functional outcomes according to Flynn score. The rate of “excellent” and “good” results was similar in both groups. Sixty-two percent of patients in the TEN group had excellent results when compared with 68% of patients in the open plating group. We found that TENs had a similar complication rate when compared with plating (18% in TEN group vs 14% in OPI group). Comparison of demographic data suggested that the TEN group had younger age, lower body weight, experienced less blood loss, and showed shorter operation time. On the contrary, patients with older age and heavier weight were found in the OPI group.

There is no consensus in the literature on the definition of a pediatric subtrochanteric femur fracture.^[2,7,8]^ Ireland and Fisher ^[2]^ proposed a definition of subtrochanteric fractures: The fracture line is located within the proximal one-fourth of the interval between the intertrochanteric region and the adductor tubercle. However, according to this definition, the accuracy of measurement is less satisfactory. Pombo and Shilt ^[7]^ recently defined the pediatric subtrochanteric femur fracture as a fracture located within 10% of the total femur length below the lesser trochanter. They created their formula on the basis of the definition of an adult's subtrochanteric femur fracture, which is a fracture that occurs within 5 cm below the lesser trochanter, and the average length of the adult femur. The difference in femur lengths at various ages was taken into account in the definition by Pombo, along with the difference in femur lengths among children at the same age. We believe that the definition of Pombo is not only accurate and practical but also easily utilized. Therefore, we used classification by Pombo in this study.

The incidence of subtrochanteric fractures in children is about 4% to 10% of all children's femoral fractures.^[8]^ In general, children who are under 5 years of age may be either treated with closed reduction and spica casting with or without traction. Children who are over 12 years of age and adolescents were preferentially treated with interlocking intramedullary nailing.^[6]^ Fractures in this region particularly are difficult to manage as a subset of femur fractures. It is challenging to obtain and maintain the reduction of the proximal fracture fragment due to the flexion, abduction, and external rotation secondary to forces from the iliopsoas, hip abductors, and external rotator muscles in the subtrochanteric region of the femur.^[6]^ Jarvis et al^[4]^ retrospectively reported that about one-fourth skeletally immature adolescents with subtrochanteric fracture had poor clinical and radiologic outcomes by nonoperative management, including fracture malalignment with more than 16° of angulation, and lower limb length shortening of the affected limb by an average of 2.6 cm. Therefore, surgical intervention with internal fixation became more popular. We hypothesize that a solid internal fixation is a rewarding option for reducing complications.

There is a large variation in the individual development of children 5 to 11 years of age, and the choice of internal fixation implants for subtrochanteric fractures is controversial. TENs are currently the most popular treatment option for femoral shaft fractures in school-age children and young adolescents.^[1,9,10]^ With the development of surgical technology, the operative indication of TEN has gradually extended to the proximal femur 1/3 and subtrochanteric fractures.^[3,11]^ However, several studies have demonstrated suboptimal results with fractures in the proximal third of the femur^[1,9]^ and length-unstable fracture patterns.^[3,12]^ In the series by Flynn et al,^[1]^ the only patient with a poor Titanium Elastic Nails Outcome Score was an 11-year-old child with a proximal, oblique comminuted fracture that healed with 15 mm of shortening and 20° of varus angulation. Ho et al^[9]^ reported a 22% complication rate with proximal third femur fractures managed with TENs. Both Narayanan et al^[12]^ and Sink et al^[3]^ reported a higher complication rate and risk of unplanned revision surgery with length-unstable femur fractures treated with TENs. Therefore, other internal fixation methods are recommended to replace TEN in the treatment of subtrochanteric fractures of the femur in children. Ellis et al^[13]^ reported that the distal femoral interlocking intramedullary nail represents a successful treatment of length unstable fractures in older children, which can effectively maintain fracture reduction and prevent occurrence of shortening. Kanlic et al^[14]^ reported a 4% complication rate in 51 pediatric femoral shaft fractures treated with submuscular plating. Twenty-four percent of the fractures were in the subtrochanteric region and 55% of the fractures were unstable. It was believed that interlocking intramedullary nail or compression plate technology had biomechanical advantages in maintaining the length and stabilization of the reduction of fractures, although they were more demanding to apply and carried a potential risk of injury to the epiphysis. A multicenter retrospective study by Ying et al^[6]^ showed that the outcome of subtrochanteric fractures treated with plating in school-age was better when compared with TEN fixation. But according to their study, open plating and submuscular plating were not analyzed separately, which may amplify the excellent results. Parikh et al^[11]^ recently demonstrated that the outcomes of 33 patients with subtrochanteric fractures in school-age treated by TEN were satisfactory. Major complications such as nonunion, malunion, or limb length discrepancy and other serious complications after operation were low. The long-term follow-up showed a good prognosis, so the authors believed that considering TEN represents a safe and efficient option to treat subtrochanteric femur fractures in children.^[11]^ Closed reduction combined with TEN fixation is less invasive when compared with open reduction and locking plate fixation. TEN fixation is our preferred method of stabilization for subtrochanteric femur fractures in younger children and children of lower body weight. Locking plate fixation provides better stability and thus results in a lower rate of complications.

The application of cast immobilization in pediatric femoral fractures especially in the proximal third of the femur is discussed controversially. Some authors believe proximal third femur fractures or length-unstable fractures treated with TEN may benefit from additional postoperative immobilization.^[1,10,12]^ However, Sink et al^[3]^ did not find that routine use of a single-leg spica cast decreased the complication rate. We usually immobilized pediatric femoral fractures by spica cast for 6 weeks until radiographic union was noted at follow-up (bridging callus across at least 3 of the 4 cortices on AP and lateral radiographs). For the OPI patients, the long cast ends just proximal above the knee and distal far from the subtrochanteric region. The function of using a long leg cast in OPI patients is to immobilize the involved limb and delay the time to weightbearing.

Limitations of this retrospective study are that the patients were not randomized to the treatment groups. Selection bias did exist in this retrospective study. Some of the surgeons who managed the patients in this study may have preferentially chosen other treatment options. The selection bias of patients is not eliminated. A prospective study design might be the best way to reduce selection bias in a further study. A second limitation is our use of the outcome score by Flynn to classify outcomes of subtrochanteric femur fractures treated with open plating. The outcome score was designed to assess the results of TEN but may not be suitable for the assessment of plating outcomes.

## 7 Conclusion

We directly compared TENs with open plating for the treatment of subtrochanteric femur fractures in school-aged children. We found that both TEN and OPI stabilization can result in good functional outcomes according to the score by Flynn. The rate of “excellent” and “good” results is similar in both groups. The ROM of hip and NSA of the affected limb were almost the same as those of the unaffected side. Demographic data suggested that TEN was more suitable for children with younger age and lower body weight, and TEN had more advantages with less blood loss and operation time. On the contrary, patients with older age and heavier weight may benefit from open plate fixation.

## Author contributions

**Conceptualization:** Kaiying Shen.

**Data curation:** Jingxia Bian, Kaiying Shen.

**Formal analysis:** Yunlan Xu, Bin Xue.

**Investigation:** Jingxia Bian, Bin Xue.

**Methodology:** Yunlan Xu, Kaiying Shen, Bin Xue.

**Software:** Kaiying Shen.

**Writing – original draft:** Yunlan Xu, Jingxia Bian.

**Writing – review & editing:** Kaiying Shen.

## References

[1] Flynn JM Hresko T Reynolds RAK Titanium elastic nails for pediatric femur fractures: a multicenter study of early results with analysis of complications. J Pediatr Orthop 2001;21:4–8. 11176345 10.1097/00004694-200101000-00003

[2] Ireland DC Fisher RL Subtrochanteric fractures of the femur in children. Clin Orthop Relat Res 1975;157–66. 1157378 10.1097/00003086-197507000-00020

[3] Sink EL Gralla J Repine M Complications of pediatric femur fractures treated with titanium elastic nails: a comparison of fracture types. J Pediatr Orthop 2005;25:577–80. 16199934 10.1097/01.bpo.0000164872.44195.4f

[4] Jarvis J Davidson D Letts M Management of subtrochanteric fractures in skeletally immature adolescents. J Trauma 2006;60:613–9. 16531863 10.1097/01.ta.0000197606.63124.9e

[5] Park KC Oh CW Byun YS Intramedullary nailing versus submuscular plating in adolescent femoral fracture. Injury 2012;43:870–5. 22154047 10.1016/j.injury.2011.10.032

[6] Ying Li Benton EH Michael G Comparison of titanium elastic nail and plate fixation of pediatric subtrochanteric femur fractures. J Pediatr Orthop 2013;33:232–8. 23482257 10.1097/BPO.0b013e318288b496

[7] Pombo MW Shilt JS The definition and treatment of pediatric subtrochanteric femur fractures with titanium elastic nails. J Pediatr Orthop 2006;26:364–70. 16670550 10.1097/01.bpo.0000203005.50906.41

[8] Jeng C Sponseller PD Yates A Subtrochanteric femoral fractures in children. Alignment after 90 degrees-90 degrees traction and cast application. Clin Orthop Relat Res 1997;170–4. 9269171

[9] Ho CA Skaggs DL Tang CW Use of flexible intramedullary nails in pediatric femur fractures. J Pediatr Orthop 2006;26:497–504. 16791069 10.1097/01.bpo.0000226280.93577.c1

[10] Luhmann SJ Schootman M Schoenecker PL Complications of titanium elastic nails for pediatric femoral shaft fractures. J Pediatr Orthop 2003;23:443–7. 12826940

[11] Parikh S Nathan S Priola M Elastic nailing for pediatric subtrochanteric and supracondylar femur fractures. Clin Orthop Relat Res 2014;472:2735–44. 23955195 10.1007/s11999-013-3240-z PMC4117889

[12] Narayanan UG Hyman JE Wainwright AM Complications of elastic stable intramedullary nail fixation of pediatric femoral fractures, and how to avoid them. J Pediatr Orthop 2004;24:363–9. 15205616 10.1097/00004694-200407000-00004

[13] Ellis HB Ho CA Podeszwa DA A comparison of locked versus nonlocked enders rods for length unstable pediatric femoral shaft fractures. J Pediatr Orthop 2011;31:825–33. 22101659 10.1097/BPO.0b013e31822ed34d

[14] Kanlic EM Anglen JO Smith DG Advantages of submuscular bridge plating for complex pediatric femur fractures. Clin Orthop Relat Res 2004;244–51. 10.1097/01.blo.0000138961.34810.af 15346081

